# Physician perceptions of the types of roles interpreters play in limited English proficient pediatric encounters and how they evaluate the quality of interpretation

**DOI:** 10.1017/S1463423618000890

**Published:** 2019-03-20

**Authors:** Rebecca J. Schwei, Natalie Guerrero, Alissa L. Small, Elizabeth A. Jacobs

**Affiliations:** 1 Assistant Researcher, Department of Medicine, University of Wisconsin-Madison School of Medicine and Public Health, Madison, WI, USA; 2 MD/PhD Student, Medical Scientist Training Program, Department of Population Health Sciences, University of Wisconsin-Madison School of Medicine and Public Health, Madison, WI, USA; 3 Medical Student, University of Wisconsin-Madison School of Medicine and Public Health, Madison, WI, USA; 4 Professor of Medicine and Population Health Sciences, Departments of Medicine and Population Health Sciences, University of Wisconsin-Madison School of Medicine and Public Health, Madison, WI, USA

**Keywords:** interpreter quality, interpreter role, language barriers, limited English proficient, pediatrics

## Abstract

**Purpose:**

The purpose of this study is to understand different roles that interpreters play in a pediatric, limited English proficient (LEP) health care encounter and to describe what factors within each role inform physicians’ assessment of the overall quality of interpretation.

**Background:**

Language barriers contribute to lower quality of care in LEP pediatric patients compared to their English-speaking counterparts. Use of professional medical interpreters has been shown to improve communication and decrease medical errors in pediatric LEP patients. In addition, in many pediatric encounters, interpreters take on roles beyond that of a pure language conduit.

**Methods:**

We conducted 11 semi-structured interviews with pediatricians and family medicine physicians in one health system. Transcripts were audio-recorded and transcribed verbatim. We analyzed our data using directed content analysis. Two study team members coded all transcripts, reviewed agreement, and resolved discrepancies.

**Findings:**

Physicians described four different interpreter roles: language conduit, flow manager, relationship builder, and cultural insider. Within each role, physicians described components of quality that informed their assessment of the overall quality of interpretation during a pediatric encounter. We found that for many physicians, a high-quality interpreted encounter involves multiple roles beyond language transmission. It is important for health care systems to understand how health care staff conceptualize these relationships so that they can develop appropriate expectations and trainings for medical interpreters in order to improve health outcomes in pediatric LEP patients.

## Introduction

In 2013, ~25.1 million individuals in the United States were considered limited English proficient (LEP), which is defined as anyone above the age of 5 who reports speaking English less than ‘very well’ (‘The Limited English Proficient Population in the United States,’ 2015). Of these 25.1 million people, 10% were children between the ages of 5 and 17 (‘The Limited English Proficient Population in the United States,’ 2015).

Previous research has documented that language barriers contribute to lower quality of care in LEP pediatric patients compared to their English-speaking counterparts (Flores *et al*., 2005; Cohen and Christakis, 2006; Galbraith *et al*., 2008; Mayo *et al*., 2016) For example, children whose parents’ primary language at home was not English less often received timely illness and routine care (Galbraith *et al*., 2008), and infants were half as likely to receive preventive care as compared to infants of parents whose primary language at home was English (Cohen and Christakis, 2006). In addition, parental LEP status is associated with triple the odds of fair/poor health status in children (Flores *et al*., 2005), and LEP parents have reported being dissatisfied with provider communication and their own lack of ability to participate effectively in decision making for their children (Mayo *et al*., 2016).

Use of professional medical interpreters has been shown to decrease health disparities in adult LEP patients (Jacobs *et al*., 2001) and improve communication and decrease medical errors in pediatric LEP patients (Flores *et al*., 2003). Traditionally, in biomedical literature, interpreter quality has been assessed based on errors in medical interpretation or measures of communication generally (Laws *et al*., 2004; Flores, 2005; Flores *et al*., 2005; Green *et al*., 2005; Gany *et al*., 2010; Jackson *et al*., 2011; Nápoles *et al*., 2015). However, in many pediatric encounters, interpreters take on roles beyond that of a pure language conduit, such as cultural broker or emotional supporter (Leanza, 2005; Abbe *et al*., 2006; White and Laws, 2009; Hsieh and Hong, 2010; Leanza *et al*., 2015; Granhagen Jungner *et al*., 2016). To better understand the ways in which interpreters mitigate health disparities in pediatric encounters, it is important to understand how the different roles that interpreters play contribute to the perception of a high-quality interpreted encounter. The purpose of this study is to understand the different roles that interpreters play in a pediatric LEP health care encounter and to describe what factors within each role inform physicians’ assessment of the overall quality of interpretation during these encounters.

## Methods

### Sample

We recruited five pediatricians and six family medicine physicians for semi-structured interviews in 2015. Physicians were part of a large health system in Wisconsin. All participants provided verbal consent, and the University of Wisconsin Institutional Review Board ruled the study exempt.

We used a number of strategies to recruit physicians. First, we sent an email to pediatricians and family physicians in our health system informing them about the study and asking them to contact the study team if they were interested. Second, the principal investigator gave presentations at provider meetings and asked interested participants to contact our study team for an interview. We excluded residents and physicians who stated that they did not have experience working with LEP patients. We invited all other physicians that responded to participate in the study. Participants received $50 in appreciation of his/her time.

### Semi-structured interview process

One or two study team members attended all interviews. Both interviewers were trained in qualitative research methods. We conducted most interviews in a private meeting room; one was conducted outside. The interview guide consisted of fourteen open-ended questions designed to promote discussion. We used probing questions to clarify or elicit a more thorough response. We asked physicians to focus on pediatric encounters where both a parent and a child were present. We audiotaped all interviews, and they were ~30 min in length. We transcribed audio files verbatim. All transcripts were reviewed for accuracy by a second researcher.

### Coding and analysis of interviews

The final sample size was determined when the study team decided that we had reached theme saturation using the constant comparison method. We proceeded with sampling, data collection and preliminary data analysis concurrently and stopped data collection when the physician responses became redundant and attempts to uncover new themes failed to reveal novel data (Bowen, 2008).

We used directed content analysis to analyze the data (Graneheim and Lundman, 2004; Hsieh and Shannon, 2005). First, study team members read through the transcripts several times and developed a preliminary codebook based on the domains of the interview questions. Next, two study team members used the preliminary codebook to code the interviews independently and capture additional key concepts. Then the two coders met to review their codes, add new codes and finalize code definitions. The complete codebook contained 91 codes. Two study team members used the complete codebook to code all transcripts. The study team continued to meet to review coding agreement and resolve discrepancies. The study team used NVivo10 to code and organize our data during the first phase of analysis.

As analysis continued, we grouped codes into broader categories based on common meaning, and identified themes and sub-themes. The study team continued to meet throughout this phase of analysis to ensure that categories were constructed in an intuitive way that accurately reflected participants’ comments. When disagreements or questions of fidelity to participants’ true meaning arose, the study team always referred back to the transcripts. We present the final themes and sub-themes regarding the relationship between interpreter role and interpreter quality in this manuscript.

## Results

We interviewed six women and five men, and the average age of physicians was 49 (range 40–61 years old). The physicians self-reported having an average LEP pediatric patient population of 22%, and this ranged from less than 5–65%. The physicians we interviewed spent an average of 4.5 half days per week in clinic.

**Figure 1:**
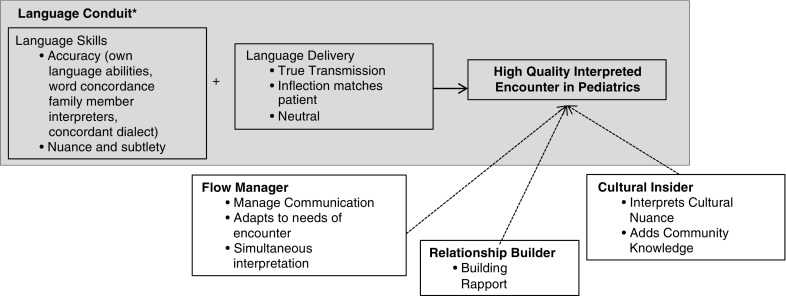
Roles interpreters play in a pediatric encounter and that factors within each role that inform providers’ assessment of interpreter quality. *Gray box highlights the interpreter role of language conduit that previous literature uses to assess quality interpretation.

Physicians described the different roles they have seen interpreters plays or roles they want interpreters to play in a medical encounter, which we describe as: language conduit, flow manager, relationship builder, and cultural insider. Figure 1 illustrates the interpreter roles physicians described and the components of quality they identified within each role that contributed to overall quality. In Figure 1, we also highlight how this work adds to the current literature; the blue box includes the components of quality that previous literature typically assessed. We will return to this in the discussion.

### Language conduit

All the physicians described that the primary role of the interpreter is as a language conduit, that is, to transmit information from one language to another. One physician described the role of a language conduit this way, ‘[The interpreters] essentially are very aware that they are facilitating a conversation.’ Within this role, physicians broadly described two separate factors: language skills and language delivery that influenced their perception of interpreter quality.

### Language skills

The physicians assessed the interpreter’s language skills, their ability to speak English and the patient’s language, based on their perception of the interpreter’s ability to communicate accurately and to communicate nuance and subtlety.

#### Accuracy

When assessing interpreters’ language skills most physicians were concerned with the accuracy of the interpretation. Physicians evaluated the accuracy of the interpretation based on their own language skills, their observations of discordant word counts, their experiences having family members as interpreters, and having concordant dialect between patient and interpreter. One physician described how she uses her Spanish language skills to assess interpreter accuracy, ‘I understand enough Spanish to know sometimes to say “wait a minute, that wasn’t what they said.” You know, and, and “what did they say about a window?” You know, I can do a little bit.’ Several physicians also described how discordant word counts were a clear signal to them that an interpreter was not accurately interpreting what was said. One physician described the discordant word count like this, ‘So you bolt out you know maybe 50 words of instructions and you, they don’t look like they’re memorizing it. And they’ll turn to the, the parent and say 10 words of instructions, and you *know* they couldn’t have possibly transmitted that.’ In addition, many physicians did not trust the accuracy of interpretation when they were using a family member as an interpreter. One physician said, ‘when they’re using a family translator, I don’t think people always translate exactly what I’m saying.’ Finally, a few physicians also commented on how accuracy may be affected when the patient and interpreter speak different dialects of a language. One physician said, ‘My understanding is that there are a number of different Arabic dialects that can really be sometimes difficult to be able to understand.’

#### Nuance and subtlety

Most physicians recognized that communication includes more than just the words that are spoken, but also includes communication through body language, the subtle choice of words or the patient’s hesitation to speak up about certain concerns. Subtleties and nuances can be lost in an interpreted encounter because the patient and the provider do not speak the same language. Therefore, many physicians described how a high-quality interpreted encounter includes an interpreter who is able to communicate subtlety and nuance. One physician described it this way, ‘So I gotta get all that subtlety and my translator’s gotta push all that information through so I can speak up to it.’ Another physician described how he witnessed an interpreter press a patient for more information when the interpreter recognized that the patient might have more to say, ‘But there were things, you know, just the nuances. [The patient] would say something and stop, and the interpreter would pick up on that, and you know, press him a little bit more.’ Generally, physicians recognized that the ability to communicate subtlety and nuance contributed to a high quality interpreted encounter.

### Language delivery

Several physicians also described language delivery, the way the interpreter interprets, as an important component of quality. Physicians described language delivery in terms of what we are calling ‘true transmission’ meaning matching inflection and neutral interpretation.

#### True transmission

Many physicians described how they wanted a true transmission of what was being said. One physician said, ‘I think we as the doctor kind of want the word-for-word interpreting.’ A second physician wanted to understand what was going on but did not feel like it always needed to be word-for-word. ‘I personally would like, I don’t need a word-by-word verbatim, kind of translation, I’m fine with the more nuanced kind of, as needed type approach that some of [the interpreters] take with people that are semi-proficient.’ Consistently, most physicians wanted a true transmission of what was being said, but many recognized that what a true transmission encompassed varied by patient language abilities.

#### Inflection

A few physicians also described how language delivery was enhanced when the interpreter’s inflection matches the patient’s inflection. One physician said, ‘What I like about her is that she will change her inflection to match the speaker. You know she could get excited or concerned to match what the speaker is saying, which I think is super cool.’ Similarly, another physician described the value of having the mannerisms and inflection interpreted:

But specific to peds, you get the mannerisms interpreted… you still get the factual, ‘these are the words I’m saying.’ But you get a lot more meaning… and we’ve got some phenomenal interpreters who will actually mimic the patient’s you know just kinda animation level and, and all of that, too.

#### Neutral interpretation

Many physicians emphasized how as a language conduit, the interpreter needs to remain neutral and leave their own thoughts and beliefs out of the conversation. One physician described an interpreter who was not able to remain neutral in an encounter. ‘This guy was kind of like an older, middle-aged guy …but he, like he would actually make comments to the young mothers about what they should do with the kids and so on.’ One physician summed up his views on interpreter neutrality in the following quote, ‘It’s incumbent on the interpreter to be extraordinarily conservative about what they say and not uh try to impose their own views on the top.’ Physicians consistently shared their belief that an interpreter needed to remain neutral in an interpreted encounter.

### Flow manager

Many physicians described how interpreters play a role in managing the flow of communication in an encounter and how this influences the quality of an interpreted encounter. Specifically, physicians described the role of flow manager in terms of the interpreter’s ability to manage communication by communicating with all parties, their ability to adapt to the needs of the encounter and their ability to do simultaneous interpretation.

### Manage communication

Several physicians described how it is important for interpreters to manage communication during an encounter and to clearly communicate with all the people in the room, so everyone understands what is being discussed. One physician said, ‘…some of the best interpreters would say to the patient, like ‘I’m just gonna tell the doctor first a little bit about that’ you know, so she knows what we’re talking about as well.’ The need to ensure all people in the room know what is being said seems to be a particularly important component of managing the flow in pediatric encounters because the children may speak English when the parents do not. In these situations, the physicians recognized that ‘then the interpreter should really be interpreting to the mom, like what the child, the child responds in English. So, the interpreter then should be [interpreting what the child said to the mom], and usually [this interpretation for the mom] doesn’t happen.’ Physicians also shared their perception that it is the interpreter’s job to check and ensure everyone understands:

I think also being able to check with the patient, ‘do you understand this? Do you, is this something that you know, are there more questions?’ You know that type of thing. And I think also in terms of making sure I’m understanding, too, so that sort of two-way check, you know… I think is really important.

### Adapts to needs of encounter

A few physicians described how the role of an interpreter can change based on the English proficiency of patients. Physicians described interpreters who are able to adapt to the language needs of the patients as higher-quality interpreters. One physician described it this way:

Sometimes the interpreters you know have a very keen understanding of [the patient’s] level of proficiency… maybe they dealt with the family multiple times, and they talk with them a little offline too you know without me, and so then they have a better understanding of what the needs are. And then sometimes they spell it out you know saying, ‘I’m here if you have any words you don’t understand, any you know issues that you don’t understand, let us know.’

However, physicians recognized that not all interpreters are able to adapt to the patient’s language needs. One physician said, ‘I had a Japanese language interpreter once who kind of just stood there and smiled and I had to kind of say, ‘Um, I need you to try to.’ Because the family from my perception looked like they were struggling a little to say things in English.’ Generally, physicians recognized an interpreter that is able to adapt to the language needs of the patients in the encounter as a higher quality interpreter compared to the interpreters who are less flexible.

### Simultaneous interpretation

Many physicians described how they wanted the interpreters to interpret simultaneously or echo their speech. One physician described how the best interpreters echo what the physician is saying:

So, the best translators I would you know, I can set a pace with, I can say you know ‘tell them he’s washing once a week, washing once a week. After the wash, do air drying, after the wash do air-drying. Um gently put on talc powder, not too aggressively, gently put on talc power, not too aggressively. You know, watch for signs of redness, watch for signs of redness.’ So they just kind of do an echo.’

In contrast, a few physicians did not like it when interpreters would wait for all of the instructions and then speak to patients. ‘The worst interpreters are the ones who, in my opinion…you’re giving a lot of detailed instructions but they don’t stop you for like ‘wait a second I need to translate some of that.’ Generally, physicians wanted language delivery to be simultaneous to their own speech.

### Approach to the patient–physician relationship

Many physicians recognized the role that interpreters have in building relationships with both the patients and providers. However, they had differing ideas regarding whether or not they wanted interpreters to take on the role of relationship builder. One provider stated, ‘The best interpreters keep a low key presence. They don’t make, they’re very observant, they don’t make a lot of eye contact.’ Another provider described how she noticed that interpreters take different approaches to building relationships with patients:

And [the interpreter’s name], she becomes very much a part of the visit… but there’s other interpreters that really are in the corner you know and they’re just kind of talking as you talk and almost like trying to get the words to you know like come through me which is good too. So, that’s two different ways of doing things. I almost like [the first] way better.

Physicians that were supportive of interpreters building relationships described how quality of care improved as interpreters built rapport with both patients and providers.

### Building rapport

Most physicians spoke about the important role that interpreters have in building rapport with patients and providers. One physician said, ‘I think you know patients, when they feel comfortable with an interpreter, I think that, that facilitates the interaction.’ Similarly, one physician described how having an existing relationship with the interpreter facilitated communication. He said, ‘We have the same cadre of interpreters available to us … I know them well; they know me well. They know some of the goofball vocabulary words I use, and it’s easy.’ Alternatively, when interpreters do not build rapport, the physicians perceive the encounter as stiff and formal. One physician said:

you know sometimes the interpreters who are just very business-like and say only what you say and don’t interact as much, or don’t have that, that kind of social connection, it seems a little, a little more you know stiff you know, I guess, or formal.

In general, when interpreters had positive rapport with patients and the physicians, the physicians perceived the encounter to be improved.

### Cultural insider

Several providers described how they appreciated when interpreters were able to add additional information to an encounter by communicating cultural nuance or by explaining experiences of the local language community.

### Cultural nuance

Some providers said that they wanted the interpreters to explain cultural nuances during LEP encounters. One provider described her desire for help with cultural nuances this way, ‘I want both, you know, to be able to, to be able to say “I do want the, you know more word for word, but if you can help me with the nuances as well would be, would be really great.’” However, physicians did not always agree interpreters should play this role. One physician said, ‘I think that’s a tricky role to play. So I don’t think people should do it unless they’re really seeing kind of some big, big breakdowns in communication.’ One physician gave the following example of a type of cultural nuance that she would appreciate having explained to her:

You know I can see if you’re, if you’re a Hmong interpreter and you have a provider getting frustrated with a family about why they’re not getting medication, you know, you might feel that it’s gonna help everyone just to say, out of the [exam] room, not necessarily about that family, but to say ‘look, just in general, I need you to know that sometimes Hmong families don’t take too well to giving medication and it’s because of a belief of this.’ You know, whatever, you know, is that outside the bounds of a traditional interpreting role? Sure it is, but who else is gonna give that information?

Of the few physicians who were open to interpreters helping to explain cultural nuance, they were all clear that explaining cultural nuance was very different from incorporating the interpreter’s own point of view into the conversation.

### Adds community knowledge

In addition to explaining cultural nuance during an encounter, a few physicians also described instances where interpreters were able to add information about a patient’s circumstance simply because the interpreter was part of the same local language community. One physician described his experience with interpreters adding additional information to an encounter in the following way, ‘And sometimes one of our interpreters after we’ll walk out of the room, will pull me aside and say, “just so you know, sometimes I see…” You know, and kind of try to fill me in on something, and I appreciate that.’

Overall, some physicians were open to the idea that interpreters could explain cultural nuance or add community knowledge to an encounter. However, this can be a very challenging task for interpreters because the interpreters need to remain neutral.

## Discussion

Overall, we found that physicians describe four broad roles that they see or would like to see interpreters play in pediatric health care encounters: language conduit, flow manager, relationship builder, and cultural insider. For many physicians a high-quality interpreted encounter involves factors from many of these diverse roles and does not solely involve language transmission.

Consistent with previous literature, physicians in our study described how a primary role of interpreters is to be a language conduit and to transmit language clearly and effectively (Flores *et al*., 2003; Laws *et al*., 2004; Nápoles *et al*., 2015). The blue box in Figure 1 highlights this understanding of high-quality interpreters as excellent language conduits. Our work adds to the existing literature because many physicians in our sample described how a high-quality interpreted encounter includes interpreters who are able to manage the flow of an encounter, are able to facilitate relationships between patients and providers, and are able to serve as a cultural insider. The area outside the blue box in Figure 1 highlights this expanded understanding of interpreter roles.

The idea that interpreters can be cultural conduits or emotional supporters has previously been described in other literature (Abbe *et al*., 2006; Hsieh & Hong, 2010). However, not all physicians in our sample embraced this expanded understanding of interpreter roles. Several providers described that they are only concerned with an interpreter’s ability to transmit information. It was unclear to us why some providers only want interpreters to be a language conduit. It could be that some providers do not have exposure to interpreters who take on additional roles in an encounter. Alternatively, providers might be concerned that expanding an interpreter role might make it more difficult for them to remain neutral. The debate on the appropriate role of interpreters in the pediatric health care encounter was reflected even in this one-institution qualitative study.

This study had several limitations. First, the goal of this qualitative study was not to create generalizable knowledge that may expand across health systems but to understand how providers in our local health care system perceive interpreter role and interpreter quality. As such, we only interviewed providers in one health care community. In addition, as our recruitment strategy was an opt-in system, it is possible that providers most interested or informed about interpreters were most interested in participating in our study. However, our analysis also has several strengths. We interviewed two different types of providers that interact with pediatric patients. In addition, the study was conducted in a system that has interpreters available for every encounter. This is a strength because it likely removes issues of access to interpreters as a concern and allows the interviewed providers to consider and discuss other components of interpreter quality.

## Conclusions

Our study supports the idea that in an LEP encounter, providers consider a wide variety of factors when considering interpreter quality. Often, these components of quality go beyond the role of being a language conduit. As the need for medical interpretation continues to rise, it is essential that health care systems clearly recognize the various roles that interpreters play in health care encounters and begin to think about what roles they do and do not want interpreters to play.

Understanding how providers conceptualize interpreter quality and how components of quality fit into the roles that interpreters play is an important first step in beginning to holistically describe the definition of high-quality medical interpreting. It is also important for health care systems to understand how patients and interpreters conceptualize a high-quality interpreted encounter. Once there is a more complete understanding of how all sides understand this issue then health care systems can identify a more accurate description of expectations and develop additional trainings for medical interpreters. This would also allow health care systems and providers the opportunity to be more explicit with interpreters regarding the roles that they do and do not want interpreters to take on in any given health care encounter. Finally, a more complete understanding of this topic will help health care providers understand the mechanisms by which the use of interpreters decreases health disparities in LEP pediatric encounters.

## Financial support

This work was supported by the University of Wisconsin Carbone Comprehensive Cancer Center (UWCCC); the National Institutes of Health (NIH; grant numbers P30 CA014520, R25 GM083252 [to N.G.], T32 GM008692 [to N.G.]), and the University of Wisconsin School of Medicine and Public Health Shapiro Summer Research Program [to A.L.S.]. The content is solely the responsibility of the authors and does not necessarily represent the official views of the NIH).

## Conflict of interest

The authors have no conflicts of interest to report.
